# Architectural and Functional Adaptations to Eccentric Training in Adolescent Volleyball Players: A Randomized Controlled Trial

**DOI:** 10.3390/sports14030092

**Published:** 2026-03-02

**Authors:** Seda Gözener Canbülbül, Bayram Ufuk Şakul

**Affiliations:** 1 Clinical Anatomy PhD Program, Anatomy Department, Graduate School of Health Sciences, Istanbul Medipol University, 34810 Istanbul, Türkiye; usakul@medipol.edu.tr; 2 Department of Physiotherapy and Rehabilitation, Faculty of Health Sciences, Bahçeşehir University, 34353 Istanbul, Türkiye

**Keywords:** triceps surae, muscle architecture, muscle strength, vertical jump performance, adolescent athletes, volleyball, eccentric exercise, ultrasonography

## Abstract

Eccentric exercise is widely used to enhance muscle strength and performance, yet its specific effects on muscle architecture and functional outcomes in adolescent athletes remain insufficiently explored. This randomized controlled trial examined the effects of an eight-week eccentric heel-drop program on triceps surae architecture, strength, and jump performance in adolescent female volleyball players. Twenty-six athletes were randomized to an exercise group (*n* = 14) or control group (*n* = 12). The exercise group performed supervised heel-drops three times weekly, while controls continued regular training. Ultrasound assessed the muscle thickness, fascicle length, and pennation angle of the gastrocnemius medialis, gastrocnemius lateralis, and soleus. Strength was measured via dynamometry, and vertical jumps (squat, countermovement, block, attack) were evaluated. The exercise group showed significant increases in gastrocnemius medialis muscle thickness (*p* = 0.03) and fascicle length (*p* = 0.002), with no changes in other muscles. While both groups improved strength, no between-group differences emerged. However, the exercise group demonstrated significant improvements in squat, block, and attack jump performance (*p* < 0.05). Consequently, eccentric plantar flexor training elicited muscle-specific architectural adaptations and improved sport-specific jump performance. Progressive eccentric heel-drop exercises serve as an effective addition to youth conditioning programs to enhance structural and functional outcomes.

## 1. Introduction

Skeletal muscle architecture, defined as the geometric arrangement of fascicles relative to the force-generating axis, determines a muscle’s capacity to produce force and movement [1,2]. Key architectural parameters—fascicle length, pennation angle, and muscle thickness—are frequently used in muscle physiology and biomechanics studies to evaluate anatomical and functional properties [3,4]. These parameters remain dynamic and are influenced by factors such as age, sex, and training status [5,6] and serve as sensitive markers of muscle adaptation to mechanical loading [7].

Fascicle length reflects the number of sarcomeres in series and is linked to shortening velocity and excursion range, whereas pennation angle represents the sarcomeres in parallel, contributing to force capacity [1,8]. Muscle thickness integrates both parameters, serving as a morphological indicator of hypertrophy and overall muscle development [7].

Eccentric contractions, characterized by active muscle lengthening under load, play a critical role in absorbing energy, modulating force, and preventing injury during dynamic movements [9,10]. High forces generated at a low metabolic cost have led to widespread use of eccentric exercises in sports and rehabilitation contexts for enhancing muscle strength and promoting structural adaptations [11].

Eccentric resistance training is known to increase fascicle length and muscle thickness, enhancing the functional capacity of lower-limb muscles [7,12]. However, the magnitude and pattern of adaptation vary across muscle groups and protocols [7,13,14]. In the triceps surae, which includes the gastrocnemius medialis, gastrocnemius lateralis, and soleus, previous studies have reported inconsistent findings regarding architectural adaptations [12,15,16]. Variability may arise from architectural and functional differences among the three muscles [4,17] or from distinct physiological characteristics of the populations studied.

Most studies on eccentric adaptations have focused on adult populations [7,10]. However, adolescence represents a critical period of morphological plasticity characterized by rapid skeletal growth and hormonal fluctuations [18]. Whether the immature musculoskeletal system of adolescent female athletes adapts to eccentric loading through mechanisms similar to those observed in adults remains insufficiently explored. Clarifying these muscle-specific adaptations holds particular importance for youth athlete development, as longer muscle fascicles have been positively associated with superior sprint and jump performance [19,20].

This study aimed to examine the effects of an 8-week eccentric training program on triceps surae muscle architecture, strength, and vertical jump performance in adolescent female volleyball players. The study hypothesized that the eccentric training protocol would induce significant increases in triceps surae muscle architecture parameters and that these adaptations would lead to improvements in vertical jump performance.

## 2. Materials and Methods

### 2.1. Study Design and Ethical Approval

This randomized controlled longitudinal study examined the effects of an 8-week eccentric training program on triceps surae muscle architecture, strength, and jump performance in adolescent female volleyball players. The study was approved by the Ethics Committee of Istanbul Medipol University (Protocol No: E-10840098-772.02-7779) and conducted in accordance with the Declaration of Helsinki. Data collection was carried out at the laboratories of Istanbul Medipol University, Istanbul, Türkiye. The study was registered at ClinicalTrials.gov (Identifier: NCT06573879). Written informed consent was obtained from all participants and their parents.

### 2.2. Participants

**Figure 1:**
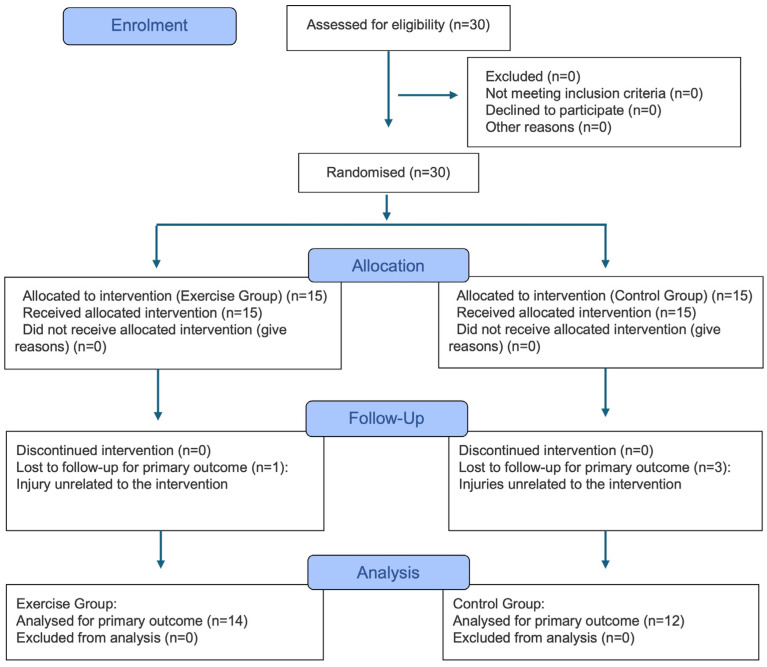
CONSORT flow diagram of the randomized controlled trial displaying participant flow through enrollment, allocation, follow-up, and analysis.

An a priori sample size calculation was performed using G*Power (version 3.1.9.4; Düsseldorf, Germany). Based on a moderate-to-large effect size (Cohen’s d = 0.60) [21] derived from a similar previous study [22], an alpha level of 0.05, and a power of 0.80, a minimum of 24 participants (12 per group) was required. Thirty healthy female volleyball players initially volunteered. Participants were recruited from the youth academies of professional volleyball clubs and actively competed in the official youth leagues. Inclusion criteria required athletes to be active volleyball players with no history of systemic disease, lower-limb injury, or orthopedic surgery in the past 6 months. Participants were randomly assigned to either the exercise group (*n* = 15) or the control group (*n* = 15) using a computer-generated randomization sequence. To prevent selection bias, allocation concealment was ensured using sequentially numbered, opaque, sealed envelopes prepared by an independent researcher who was not involved in the recruitment or assessment of the participants. During the study period, four participants withdrew due to injuries unrelated to the intervention, leaving 26 athletes for the final analysis (exercise group: *n* = 14; control group: *n* = 12) (Figure 1; see also File S1 for the CONSORT Checklist). The overall demographic characteristics of the analyzed participants were as follows (mean ± standard deviation [SD]): age 17.2 ± 1.1 years, height 177 ± 5.9 cm, and weight 66.6 ± 7.8 kg.

### 2.3. Eccentric Exercise Program

**Figure 2:**
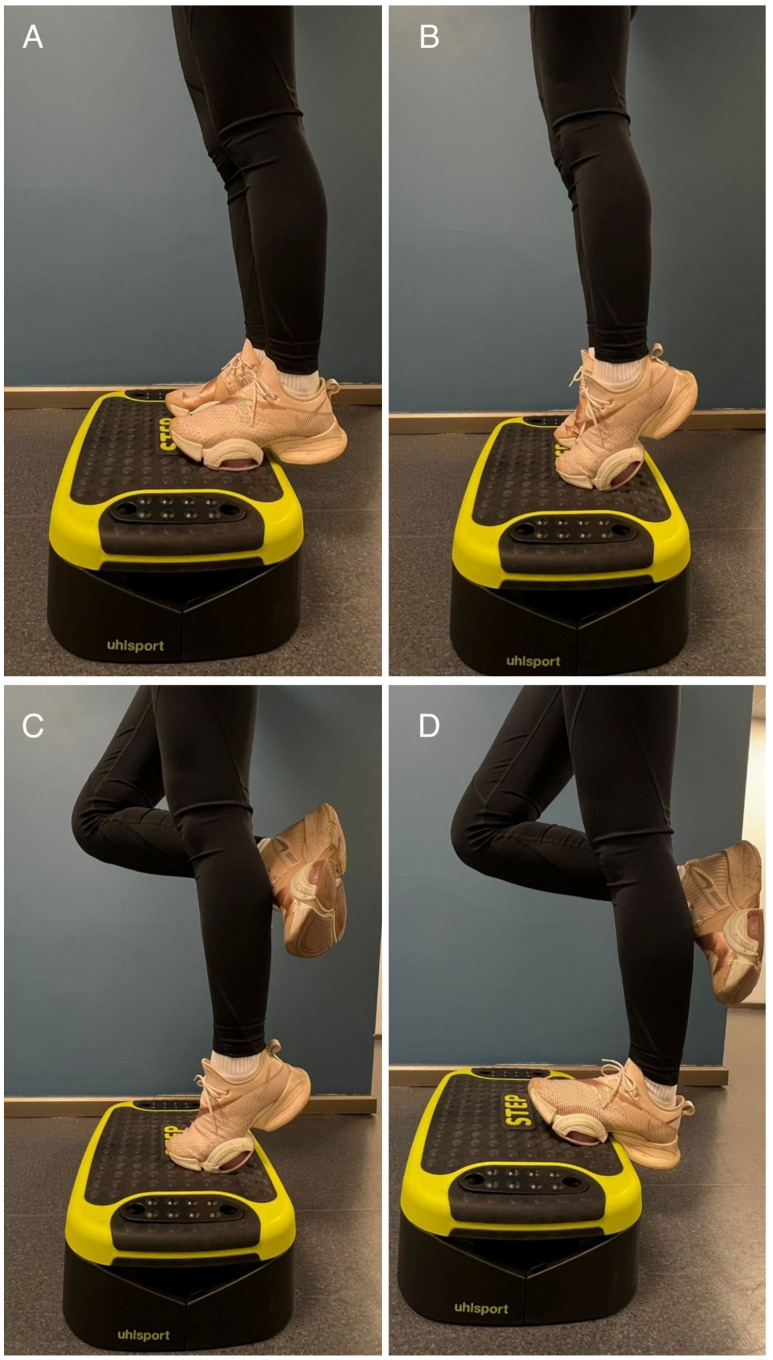
Eccentric gastrocnemius exercise. (**A**) Starting position. (**B**) Bilateral heel raise. (**C**) Non-working leg lifted. (**D**) Lowering the heel of the working leg.

**Figure 3:**
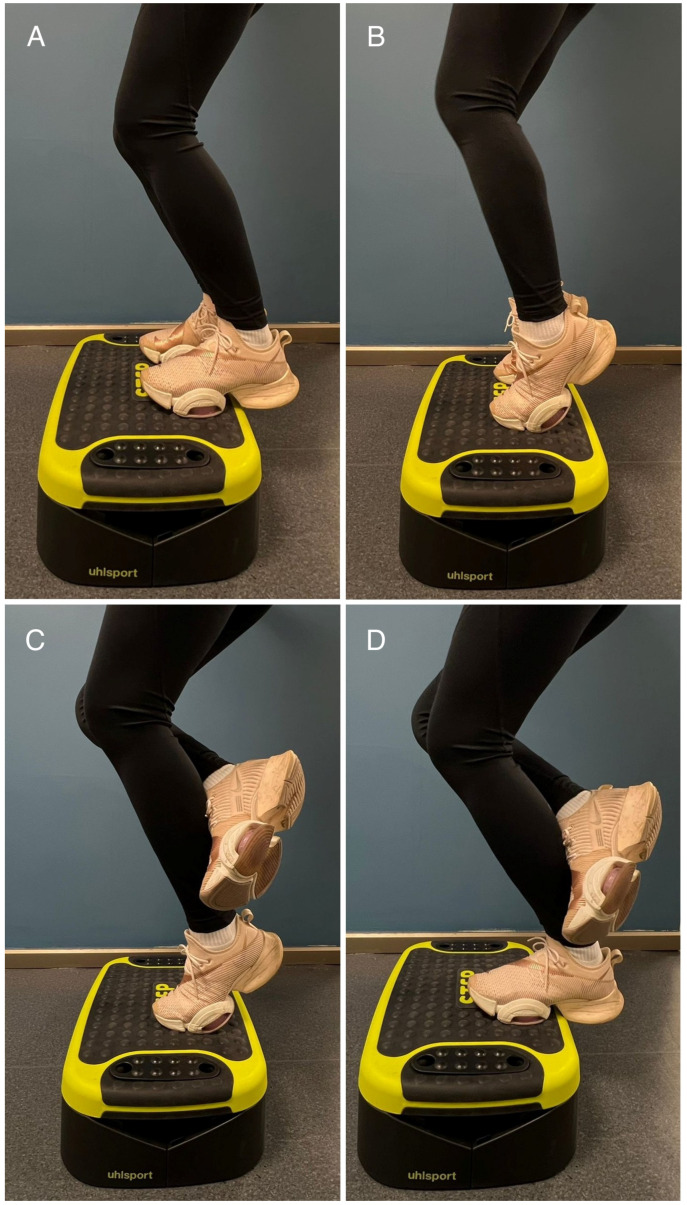
Eccentric soleus exercise. (**A**) Starting position. (**B**) Bilateral heel raise. (**C**) Non-working leg lifted. (**D**) Lowering the heel of the working leg.

The exercise group performed an 8-week supervised eccentric “heel-drop” training program targeting the triceps surae, conducted three times per week on non-consecutive days. The protocol consisted of two exercise variations: (1) knee extended to emphasize the gastrocnemius (Figure 2) and (2) knee flexed (~45°) to target the soleus (Figure 3) [23]. Each session comprised three sets of 10 repetitions performed unilaterally on the dominant leg. To ensure safety and maintain proper technique in this adolescent population, training load was prescribed using the 10-repetition maximum method [24]. The initial training load was set at the participants’ 10-repetition maximum load [25,26]. The load was increased by 5% every 2 weeks, provided the participant could complete all repetitions with proper technique. A physiotherapist supervised all sessions. Both groups continued their regular volleyball training routine, which consisted of three sessions per week, with each session lasting approximately 90 min. Standard training sessions included a standardized warm-up (jogging and dynamic stretching), technical drills (serving, passing, setting), plyometric exercises (jumping series, blocking/spiking drills), and tactical matchplay. The overall volleyball training load and duration were strictly matched and monitored by the team’s coaching staff to ensure equal exposure to court time and drills for all participants. The control group continued regular volleyball training without additional eccentric loading. Post-intervention assessments were conducted at least 48 h after the final training session.

### 2.4. Assessments

All measurements and subsequent image analyses were performed at baseline and post-intervention by the same examiner. To ensure complete blinding, all participant information was concealed, and ultrasound images were de-identified and assigned coded numbers prior to analysis. Thus, the examiner was completely blinded to the participants’ identities and group allocations.

#### 2.4.1. Muscle Architecture

**Figure 4:**
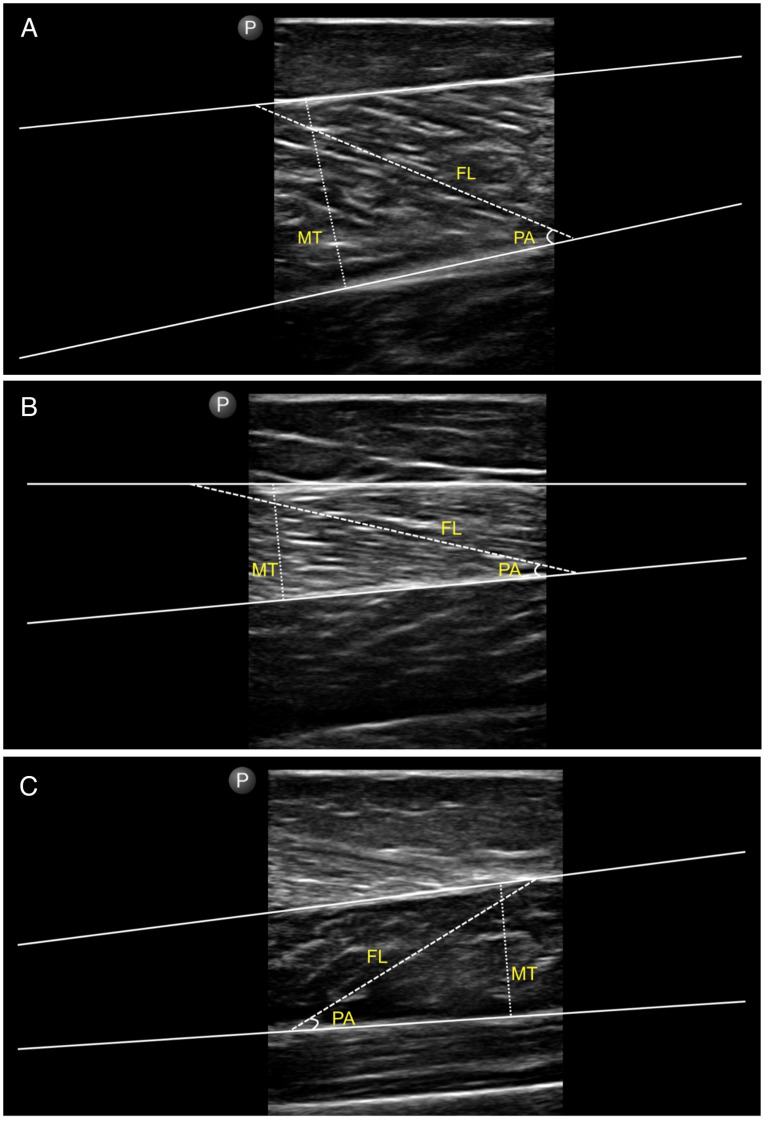
Ultrasound measurement of triceps surae muscle architecture. Representative ultrasound images of the (**A**) gastrocnemius medialis, (**B**) gastrocnemius lateralis, and (**C**) soleus. Superficial and deep aponeuroses appear as continuous lines. Fascicle length (FL) and muscle thickness (MT) were measured between the aponeuroses, and pennation angle (PA) was defined as the angle between the fascicle and the deep aponeurosis.

B-mode ultrasound imaging (Philips Lumify L12-4 MHz, Amsterdam, The Netherlands) was used to evaluate fascicle length, pennation angle, and muscle thickness of the gastrocnemius medialis, gastrocnemius lateralis, and soleus [3,4]. Participants were positioned prone with the knee flexed at 30° and the ankle in a neutral position (90°). Three longitudinal images were captured for each muscle at standardized anatomical landmarks [27,28]. Images were analyzed using ImageJ software (version 1.48v; NIH, Bethesda, MD, USA). Fascicle length was defined as the linear distance between the superficial and deep aponeuroses; when the entire fascicle was not visible, fascicle length was estimated using linear extrapolation [29]. Pennation angle was measured as the angle between the muscle fascicle and the deep aponeurosis. Muscle thickness was determined as the perpendicular distance between the two aponeuroses. The mean of three measurements was used for statistical analysis (Figure 4).

#### 2.4.2. Muscle Strength

Isometric plantar flexion strength was assessed using a hand-held dynamometer (Commander Powertrack, JTECH Medical, Midvale, UT, USA). To distinguish the relative contributions of the triceps surae components, testing was performed in two positions: (1) prone with knees fully extended (0°) to emphasize the gastrocnemius, and (2) prone with knees flexed at 30° to reduce gastrocnemius involvement and isolate the soleus [30]. Participants completed three maximal voluntary isometric contractions of 5 s each, with 30 s rest intervals between attempts. The mean of the three trials was recorded for analysis.

#### 2.4.3. Functional Performance

Vertical jump performance was evaluated using an optical measurement system (Optojump, Microgate, Bolzano, Italy) [31]. Following a standardized 5 min warm-up, participants performed four jump types: squat jump, countermovement jump, block jump, and attack jump. Three valid trials were collected for each jump type, with 3–5 min of rest between attempts. The squat jump began from a static position with 90° of knee flexion. The countermovement jump involved a rapid downward and upward movement with arm swing. The block jump replicated the volleyball blocking action, and the attack jump included an approach run, with only the vertical component evaluated [32].

### 2.5. Statistical Analysis

All statistical analyses were conducted using Jamovi software (version 2.5.6) [33]. Data normality was examined using the Shapiro–Wilk test. Paired sample t-tests were used to assess within-group changes (pre- vs. post-training). Between-group differences in adaptation were assessed using independent *t*-tests on absolute change scores (post- minus pre-value). Effect sizes (Cohen’s d) were calculated and classified as small (0.20–0.50), medium (0.51–0.80), or large (>0.81) [21]. Statistical significance was set at *p* < 0.05.

## 3. Results

Thirty athletes were enrolled, and 26 completed the study (exercise group: *n* = 14; control group: *n* = 12). No significant baseline differences were observed between groups for age, height, body mass, or training experience (*p* > 0.05) (exercise: 16.86 ± 0.95 years, 175.21 ± 5.44 cm, 63.93 ± 6.97 kg, 7.71 ± 1.14 years; control: 17.58 ± 1.16 years, 179.58 ± 5.92 cm, 69.67 ± 7.85 kg, 8.75 ± 1.60 years, respectively) (Table 1).

### 3.1. Muscle Architecture

**Figure 5:**
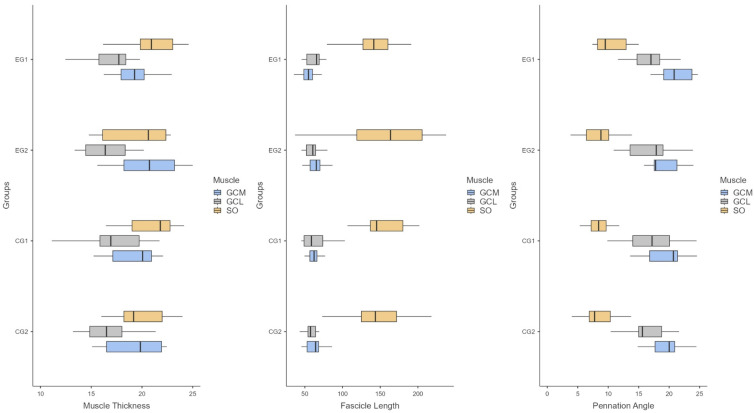
Changes in muscle thickness, fascicle length, and pennation angle of the triceps surae before and after the 8-week intervention in the exercise and control groups. Boxes represent the distribution (median, quartiles, and range). EG, exercise group; CG, control group; GCM, gastrocnemius medialis; GCL, gastrocnemius lateralis; SO, soleus.

The exercise group demonstrated significant increases in gastrocnemius medialis muscle thickness (*p* = 0.03, d = 0.67) and fascicle length (*p* = 0.002, d = 1.04) following the 8-week program. Pennation angle showed a non-significant trend toward improvement (*p* = 0.07). No significant within-group changes were observed for the gastrocnemius lateralis or soleus (*p* > 0.05). Between-group comparisons of change scores did not reveal significant differences for any architectural parameter (*p* > 0.05) (Table 2, Figure 5).

### 3.2. Muscle Strength

Both groups demonstrated significant increases in gastrocnemius and soleus strength. In the exercise group, gastrocnemius strength increased from 215 ± 17.5 N to 268 ± 47.5 N (*p* = 0.005, d = 0.90), and soleus strength increased from 189 ± 20.7 N to 246 ± 37.8 N (*p* < 0.001, d = 1.24). Similarly, the control group exhibited substantial strength gains in both the gastrocnemius (from 209 ± 19.4 N to 268 ± 24.2 N; *p* < 0.001, d = 2.10) and soleus (from 186 ± 25.4 N to 258 ± 28.6 N; *p* < 0.001, d = 3.68). Between-group comparisons revealed no significant differences in the magnitude of strength improvement for either the gastrocnemius (*p* = 0.77) or soleus (*p* = 0.31) (Table 3).

### 3.3. Vertical Jump Performance

The exercise group showed significant improvements in squat jump, block jump, and attack jump performance. Squat jump improved from 25.2 ± 2.75 cm to 27.2 ± 3.48 cm (*p* = 0.002, d = 1.05), block jump from 28.1 ± 3.12 cm to 30.1 ± 4.46 cm (*p* = 0.01, d = 0.76), and attack jump from 34.7 ± 5.35 cm to 35.8 ± 5.87 cm (*p* = 0.04, d = 0.63). Countermovement jump performance showed an improvement trend that did not reach statistical significance (*p* = 0.08). No significant changes were observed in the control group for any jump test. Between-group comparisons did not demonstrate statistical significance (*p* > 0.05); however, moderate effect sizes favoring the exercise group were observed for the block jump (*p* = 0.06, d = 0.78), countermovement jump (*p* = 0.08, d = 0.71), and squat jump (*p* = 0.09, d = 0.70) (Table 4).

## 4. Discussion

This study examined the effects of an 8-week eccentric exercise program on triceps surae muscle architecture, strength, and vertical jump performance in adolescent female volleyball players. The findings demonstrated distinct architectural adaptations in the gastrocnemius medialis, characterized by significant increases in fascicle length and muscle thickness, whereas the gastrocnemius lateralis and soleus showed no significant architectural changes. Although both groups exhibited comparable strength gains, the exercise group achieved greater improvements in squat, block, and attack jump performance.

The increases in fascicle length and muscle thickness observed in the gastrocnemius medialis align with previous studies reporting similar adaptations following eccentric training [7,15,34,35,36]. These results support the concept that eccentric contractions stimulate sarcomerogenesis, leading to the addition of sarcomeres in series; thereby increasing fascicle length [13,37]. Mechanical strain induced by eccentric loading may trigger repair processes that promote structural remodeling through serial sarcomere addition [38]. The progressive overload protocol and controlled heel-drop exercises used in this study likely enhanced this response. Previous research has also shown that eccentric and stretch-based stimuli can lengthen fascicles through similar mechanical tension mechanisms [39,40].

Functionally, longer fascicles contribute to greater shortening velocity and power output, which can translate into improved sprinting and jumping performance [2,5]. Athletes with longer fascicles demonstrate superior sprint capacity, and positive associations between fascicle length and sprint performance have been reported in elite sprinters [19,20]. Findings by Panidi et al. (2021) also showed that stretching increased fascicle length and improved jump performance in young athletes, reinforcing the adaptability of muscle architecture during adolescence [18].

No significant change in pennation angle was detected, consistent with studies showing variable or minimal effects following eccentric training [7,16]. Pennation angle adaptations may depend on training duration, contraction type, or measurement sensitivity [36,41]. Because pennation angle reflects parallel sarcomere addition [8,42], responses to eccentric loading may emerge more slowly or may occur heterogeneously across muscle regions compared to fascicle lengthening.

Selective adaptation in the gastrocnemius medialis aligns with prior evidence showing muscle-specific responses within the triceps surae [17,41]. Previous literature suggests that the gastrocnemius medialis may experience higher strain and activation during eccentric plantar flexion, whereas the gastrocnemius lateralis is hypothesized to contribute more to ankle stabilization and proprioceptive control [17,43]. The soleus, dominated by type I fibers and a multipennate structure, adapts more gradually and may require longer interventions [44,45]. Moreover, it has been shown to exhibit a blunted protein synthesis response to resistance exercise compared to other lower limb muscles [46]. Furthermore, very recent evidence suggests that the soleus may have a higher volume threshold for hypertrophy, requiring significantly higher weekly set volumes to elicit structural changes comparable to the gastrocnemius [47]. A compartmentalized neuromuscular structure may also explain the absence of measurable morphological change in the soleus in this study [48,49].

Both groups exhibited significant increases in muscle strength, with no between-group differences. Frequent jumping and landing tasks in volleyball may contribute to strength gains through sport-specific loading, as high-volume sport participation can independently enhance lower-limb strength and neuromuscular coordination [50]. An already elevated baseline training stimulus in these adolescent athletes may account for the absence of an additional effect on isometric strength in the exercise group [51].

However, the exclusive enhancements in jump performance suggest that the eccentric intervention elicited distinct neural and coordination-related adaptations. Eccentric training provides a unique neuromuscular stimulus that not only improves elastic energy storage in the stretch–shortening cycle [52]. but also alters motor unit recruitment strategies, including the preferential activation of high-threshold type II muscle fibers [51]. These targeted neural mechanisms likely potentiated concentric force output during explosive tasks, driving the superior squat, block, and attack jump performances observed in the exercise group [10,53].

The absence of significant improvement in countermovement jump height may relate to the greater involvement of hip and knee extensors in this task [54]. As the intervention primarily targeted the ankle plantar flexors, transfer to multi-joint tasks may have been limited. Additionally, this lack of transfer may stem from differences in task-specific mechanical characteristics. The CMJ relies on specific joint angular velocities, rapid stretch-shortening cycle dynamics, and distinct ground contact times that differ substantially from the controlled, slower nature of the eccentric heel-drop exercise. According to the principle of velocity specificity, resistance training adaptations are most successfully transferred to athletic performance when the training velocities and mechanical demands closely match the explosive task [55,56]. Meta-analyses support this interpretation, showing that combined eccentric and plyometric programs yield superior outcomes in countermovement jump performance compared to isolated eccentric training [57,58].

The improvements in squat, block, and attack jump performance highlight the importance of eccentric plantar flexor training for volleyball-specific movements. Previous research has shown that jump performance, particularly attack jump height, strongly correlates with offensive effectiveness in volleyball [59]. Therefore, the eccentric heel-drop protocol used in this study may contribute to improving and sustaining jump performance during match play [60].

Although the exercise group showed significant within-group improvements in jump performance that were not observed in the control group, the lack of statistical significance in between-group comparisons requires a cautious interpretation of effectiveness. It is important to note that the control group also exhibited substantial strength gains, likely due to the high volume of plyometric actions (jumping, landing) inherent in their regular volleyball training during the competitive season. This high baseline activity may have masked the additional benefits of the eccentric intervention regarding absolute strength, highlighting that eccentric training provided specific architectural and functional optimization rather than gross strength dominance over regular sport practice.

Finally, the present study is subject to certain limitations. Although the final sample size (*n* = 26) satisfied the a priori power analysis requirements for the primary outcomes, the relatively small and slightly unequal group sizes (exercise: *n* = 14, control: *n* = 12) may have reduced the statistical power to detect moderate between-group effects, particularly for strength outcomes and some architectural parameters. Furthermore, because the exercise group performed additional eccentric training, they inherently experienced a slightly higher total weekly training volume than the control group. Additionally, although randomization was successful (*p* > 0.05), the control group displayed a trend toward being slightly older and having more training experience. While this creates a potential confounding factor, current pediatric exercise science literature suggests that structural adaptations (e.g., hypertrophy) are generally more pronounced in more mature athletes due to increased hormonal availability [61,62,63]. Therefore, the fact that the relatively younger exercise group achieved significant architectural adaptations—which are typically harder to induce in less mature athletes compared to neural adaptations—reinforces the specific efficacy of the eccentric heel-drop intervention.

Moreover, specific intra-rater reliability metrics (e.g., intraclass correlation coefficients) for the ultrasound measurements were not calculated for the current sample. However, to minimize measurement error and ensure consistency, all image acquisitions and analyses were performed by a single blinded examiner strictly adhering to standardized anatomical landmarks, and the mean of three images was used for all statistical analyses. Furthermore, because the sample consisted exclusively of adolescent female volleyball players, caution should be exercised when generalizing these findings to male athletes, adult populations, or other sporting disciplines. Future research should also explore the combined effects of eccentric loading and sports nutrition or supplementation strategies, as these factors can independently influence structural adaptations and lower-limb explosive power in volleyball players [64,65].

## 5. Conclusions

This study demonstrated that an 8-week eccentric training program targeting the triceps surae in adolescent female volleyball players produced architectural adaptations in the gastrocnemius medialis and generated significant improvements in squat, block, and attack jump performance. These findings suggest that eccentric loading of the plantar flexors can enhance muscle architecture and contribute to improved explosive lower-limb performance. Future research should consider longitudinal designs with longer intervention periods (>8 weeks) to fully elucidate the time-course of adaptations, particularly for the soleus muscle. Additionally, investigating these adaptations across different stages of biological maturation would provide deeper insights into the optimal timing for implementing eccentric training in youth athletes.

## 6. Practical Implications

Incorporating progressive eccentric heel-drop exercises targeting the triceps surae into volleyball training programs for adolescent female athletes may promote architectural adaptations in the dominant gastrocnemius medialis and support improvements in jump performance. These exercises are simple to implement, require minimal equipment and time, and can be integrated into routine team conditioning sessions to enhance functional performance in adolescent players.

## Figures and Tables

**Table 1:** Baseline demographic characteristics of the participants.

Variable	Exercise Group (*n* = 14)	Control Group (*n* = 12)	*p*
Age (years)	16.86 ± 0.95	17.58 ± 1.16	0.092
Height (cm)	175.21 ± 5.44	179.58 ± 5.92	0.062
Body Mass (kg)	63.93 ± 6.97	69.67 ± 7.85	0.060
Training Experience (years)	7.71 ± 1.14	8.75 ± 1.60	0.067

Data are presented as mean ± standard deviation. Independent samples *t*-test was used to assess differences between groups.

**Table 2:** Within-group and between-group comparisons of muscle architecture parameters in the exercise and control groups.

		EG				CG				Between Groups		
Muscle	Parameter	Pre-Test	Post-Test	*p*	d	Pre-Test	Post-Test	*p*	d	MD	*p*	d
GCM	MT (mm)	19.28 ± 2.02	20.94 ± 3.09	0.03 *	−0.67	19.75 ± 2.82	19.33 ± 2.77	0.65	0.14	2.078	0.07	0.75
	FL (mm)	54.4 ± 10.35	64.3 ± 15.49	0.002 *	−1.04	61.40 ± 10.14	62.36 ± 11.18	0.80	−0.08	8.886	0.05	0.80
	PA (°)	21.9 ± 3.33	20.8 ± 3.70	0.07	0.53	20.46 ± 3.97	20.12 ± 3.70	0.80	0.08	−0.732	0.59	−0.22
GCL	MT (mm)	17.15 ± 2.71	16.42 ± 2.21	0.35	0.26	17.34 ± 3.02	16.63 ± 2.46	0.53	0.19	−0.024	0.99	−0.01
	FL (mm)	62.27 ± 11.33	64.06 ± 18.14	0.76	−0.09	64.19 ± 18.53	62.53 ± 15.36	0.73	0.10	3.451	0.65	0.18
	PA (°)	17.01 ± 3.05	17.30 ± 4.28	0.80	−0.07	17.16 ± 4.33	18.09 ± 4.65	0.58	−0.17	−0.636	0.74	−0.13
SO	MT (mm)	23.4 ± 3.82	21.9 ± 4.31	0.36	0.25	22.18 ± 3.28	20.43 ± 2.96	0.1	0.52	0.241	0.90	0.05
	FL (mm)	135.3 ± 41.58	155.1 ± 62.02	0.21	−0.36	160.04 ± 45.62	154.32 ± 63.85	0.80	0.08	25.557	0.33	0.39
	PA (°)	11.0 ± 4.03	10.2 ± 6.15	0.59	0.15	8.36 ± 1.93	8.61 ± 3.08	0.79	−0.08	−1.034	0.56	−0.23

Data are presented as mean ± standard deviation. EG, exercise group; CG, control group; MT, muscle thickness; FL, fascicle length; PA, pennation angle; GCM, gastrocnemius medialis; GCL, gastrocnemius lateralis; SO, soleus; d, Cohen’s d; MD, mean difference between groups; * *p* < 0.05.

**Table 3:** Within-group and between-group comparisons of gastrocnemius and soleus muscle strength.

	EG				CG				Between Groups		
Muscle	Pre-Test	Post-Test	*p*	d	Pre-Test	Post-Test	*p*	d	MD	*p*	d
GC	215 ± 17.5	268 ± 47.5	0.005 *	−0.90	209 ± 19.4	268 ± 24.2	<0.001 *	−2.10	−5.57	0.77	−0.19
SO	189 ± 20.7	246 ± 37.8	<0.001 *	−1.24	186 ± 25.4	258 ± 28.6	<0.001 *	−3.68	−14.82	0.31	−0.41

Data are presented as mean ± standard deviation. Muscle strength was measured in Newtons (N). EG, exercise group; CG, control group; GC, gastrocnemius; SO, soleus; d, Cohen’s d; MD, mean difference between groups; * *p* < 0.05.

**Table 4:** Within-group and between-group comparisons of vertical jump performance.

	EG				CG				Between Groups		
VJ	Pre-Test	Post-Test	*p*	d	Pre-Test	Post-Test	*p*	d	MD	*p*	d
AJ	34.7 ± 5.35	35.8 ± 5.87	0.04 *	−0.63	35.8 ± 7.59	35.1 ± 5.72	0.73	0.10	1.72	0.35	0.38
BJ	28.1 ± 3.12	30.1 ± 4.46	0.01 *	−0.76	28.5 ± 4.61	27.2 ± 5.14	0.43	0.24	3.28	0.06	0.78
CMJ	29.9 ± 4.14	31.2 ± 3.84	0.08	−0.51	30.7 ± 4.79	29.6 ± 3.40	0.37	0.268	2.38	0.08	0.71
SJ	25.2 ± 2.75	27.2 ± 3.48	0.002 *	−1.05	25.0 ± 4.59	25.4 ± 4.42	0.61	−0.151	1.57	0.09	0.70

Data are presented as mean ± standard deviation. EG, exercise group; CG, control group; AJ, attack jump; BJ, block jump; CMJ, countermovement jump; SJ, squat jump; VJ, vertical jump; d, Cohen’s d; MD, mean difference between groups; * *p* < 0.05.

## Data Availability

The raw data supporting the conclusions of this article will be made available by the authors, without undue reservation.

## Supplementary Materials

The following supporting information can be downloaded at: https://www.mdpi.com/article/10.3390/sports14030092/s1, File S1: CONSORT Checklist.

## References

[1] Fukunaga T., Ichinose Y., Ito M., Kawakami Y., Fukashiro S. (1997). Determination of Fascicle Length and Pennation in a Contracting Human Muscle in Vivo. J. Appl. Physiol. (1985).

[2] Lieber R.L., Fridén J. (2000). Functional and Clinical Significance of Skeletal Muscle Architecture. Muscle Nerve.

[3] Narici M.V., Binzoni T., Hiltbrand E., Fasel J., Terrier F., Cerretelli P. (1996). In Vivo Human Gastrocnemius Architecture with Changing Joint Angle at Rest and during Graded Isometric Contraction. J. Physiol..

[4] Maganaris C.N., Baltzopoulos V., Sargeant A.J. (1998). In Vivo Measurements of the Triceps Surae Complex Architecture in Man: Implications for Muscle Function. J. Physiol..

[5] Blazevich A.J. (2006). Effects of Physical Training and Detraining, Immobilisation, Growth and Aging on Human Fascicle Geometry. Sports Med..

[6] Martin-Rodriguez S., Gonzalez-Henriquez J.J., Diaz-Conde J.C., Calbet J.A.L., Sanchis-Moysi J. (2024). The Relationship between Muscle Thickness and Pennation Angle Is Mediated by Fascicle Length in the Muscles of the Lower Extremities. Sci. Rep..

[7] Baroni B.M., Geremia J.M., Rodrigues R., De Azevedo Franke R., Karamanidis K., Vaz M.A. (2013). Muscle Architecture Adaptations to Knee Extensor Eccentric Training: Rectus Femoris vs. Vastus Lateralis. Muscle Nerve.

[8] Kawakami Y., Ichinose Y., Fukunaga T. (1998). Architectural and Functional Features of Human Triceps Surae Muscles during Contraction. J. Appl. Physiol. (1985).

[9] Lindstedt S.L., LaStayo P.C., Reich T.E. (2001). When Active Muscles Lengthen: Properties and Consequences of Eccentric Contractions. Physiology.

[10] Franchi M.V., Reeves N.D., Narici M.V. (2017). Skeletal Muscle Remodeling in Response to Eccentric vs. Concentric Loading: Morphological, Molecular, and Metabolic Adaptations. Front. Physiol..

[11] Gash M.C., Kandle P.F., Murray I.V., Varacallo M.A. (2025). Physiology, Muscle Contraction. StatPearls.

[12] Geremia J.M., Baroni B.M., Bobbert M.F., Bini R.R., Lanferdini F.J., Vaz M.A. (2018). Effects of High Loading by Eccentric Triceps Surae Training on Achilles Tendon Properties in Humans. Eur. J. Appl. Physiol..

[13] Butterfield T.A., Leonard T.R., Herzog W. (2005). Differential Serial Sarcomere Number Adaptations in Knee Extensor Muscles of Rats Is Contraction Type Dependent. J. Appl. Physiol. (1985).

[14] Blazevich A.J., Cannavan D., Coleman D.R., Horne S. (2007). Influence of Concentric and Eccentric Resistance Training on Architectural Adaptation in Human Quadriceps Muscles. J. Appl. Physiol. (1985).

[15] Duclay J., Martin A., Duclay A., Cometti G., Pousson M. (2009). Behavior of Fascicles and the Myotendinous Junction of Human Medial Gastrocnemius Following Eccentric Strength Training. Muscle Nerve.

[16] Fouré A., Nordez A., Cornu C. (2013). Effects of Eccentric Training on Mechanical Properties of the Plantar Flexor Muscle-Tendon Complex. J. Appl. Physiol. (1985).

[17] Crouzier M., Lacourpaille L., Nordez A., Tucker K., Hug F. (2018). Neuromechanical Coupling within the Human *Triceps Surae* and Its Consequence on Individual Force Sharing Strategies. J. Exp. Biol..

[18] Panidi I., Bogdanis G.C., Terzis G., Donti A., Konrad A., Gaspari V., Donti O. (2021). Muscle Architectural and Functional Adaptations Following 12-Weeks of Stretching in Adolescent Female Athletes. Front. Physiol..

[19] Kumagai K., Abe T., Brechue W.F., Ryushi T., Takano S., Mizuno M. (2000). Sprint Performance Is Related to Muscle Fascicle Length in Male 100-m Sprinters. J. Appl. Physiol. (1985).

[20] Abe T., Fukashiro S., Harada Y., Kawamoto K. (2001). Relationship Between Sprint Performance and Muscle Fascicle Length in Female Sprinters. J. Physiol. Anthropol..

[21] Cohen J. (2013). Statistical Power Analysis for the Behavioral Sciences.

[22] Longo S., Cè E., Bisconti A.V., Rampichini S., Doria C., Borrelli M., Limonta E., Coratella G., Esposito F. (2021). The Effects of 12 Weeks of Static Stretch Training on the Functional, Mechanical, and Architectural Characteristics of the Triceps Surae Muscle–Tendon Complex. Eur. J. Appl. Physiol..

[23] Alfredson H., Pietilä T., Jonsson P., Lorentzon R. (1998). Heavy-Load Eccentric Calf Muscle Training for the Treatment of Chronic Achilles Tendinosis. Am. J. Sports Med..

[24] Faigenbaum A.D., Kraemer W.J., Blimkie C.J.R., Jeffreys I., Micheli L.J., Nitka M., Rowland T.W. (2009). Youth Resistance Training: Updated Position Statement Paper From the National Strength and Conditioning Association. J. Strength Cond. Res..

[25] Wernbom M., Augustsson J., Thomeé R. (2007). The Influence of Frequency, Intensity, Volume and Mode of Strength Training on Whole Muscle Cross-Sectional Area in Humans. Sports Med..

[26] Baechle T.R., Earle R.W., National Strength & Conditioning Association (2008). Essentials of Strength Training and Conditioning.

[27] Abellaneda S., Guissard N., Duchateau J. (2009). The Relative Lengthening of the Myotendinous Structures in the Medial Gastrocnemius during Passive Stretching Differs among Individuals. J. Appl. Physiol. (1985).

[28] Geremia J.M., Baroni B.M., Bini R.R., Lanferdini F.J., De Lima A.R., Herzog W., Vaz M.A. (2019). Triceps Surae Muscle Architecture Adaptations to Eccentric Training. Front. Physiol..

[29] Finni T., Ikegawa S., Komi P.V. (2001). Concentric Force Enhancement during Human Movement. Acta Physiol. Scand..

[30] Kovács B., Csala D., Yang S., Tihanyi J., Gu Y., Hortobágyi T. (2024). Knee Position Affects Medial Gastrocnemius and Soleus Activation during Dynamic Plantarflexion: No Evidence for an Inter-Muscle Compensation in Healthy Young Adults. Biol. Open.

[31] Glatthorn J.F., Gouge S., Nussbaumer S., Stauffacher S., Impellizzeri F.M., Maffiuletti N.A. (2011). Validity and Reliability of Optojump Photoelectric Cells for Estimating Vertical Jump Height. J. Strength Cond. Res..

[32] Sattler T., Sekulic D., Hadzic V., Uljevic O., Dervisevic E. (2012). Vertical Jumping Tests in Volleyball: Reliability, Validity, and Playing-Position Specifics. J. Strength Cond. Res..

[33] The Jamovi Project jamovi.

[34] Blazevich A.J., Sharp N.C.C. (2005). Understanding Muscle Architectural Adaptation: Macro- and Micro-Level Research. Cells Tissues Organs.

[35] Timmins R.G., Ruddy J.D., Presland J., Maniar N., Shield A.J., Williams M.D., Opar D.A. (2016). Architectural Changes of the Biceps Femoris Long Head after Concentric or Eccentric Training. Med. Sci. Sports Exerc..

[36] Lizama-Pérez R., Chirosa-Rios I., Chirosa-Rios L., Olave E., Ferragut C., Vila H., Jerez-Mayorga D. (2022). Effects of Eccentric Exercise on Muscle Architecture in Adults: A Systematic Review. Int. J. Morphol..

[37] Lynn R., Talbot J.A., Morgan D.L. (1998). Differences in Rat Skeletal Muscles after Incline and Decline Running. J. Appl. Physiol. (1985).

[38] Proske U., Morgan D.L. (2001). Muscle Damage from Eccentric Exercise: Mechanism, Mechanical Signs, Adaptation and Clinical Applications. J. Physiol..

[39] Reeves N.D., Maganaris C.N., Longo S., Narici M.V. (2009). Differential Adaptations to Eccentric versus Conventional Resistance Training in Older Humans. Exp. Physiol..

[40] Freitas S.R., Mil-Homens P. (2015). Effect of 8-Week High-Intensity Stretching Training on Biceps Femoris Architecture. J. Strength Cond. Res..

[41] Simpson C.L., Kim B.D.H., Bourcet M.R., Jones G.R., Jakobi J.M. (2017). Stretch Training Induces Unequal Adaptation in Muscle Fascicles and Thickness in Medial and Lateral Gastrocnemii. Scand. Med. Sci. Sports.

[42] Schoenfeld B.J. (2010). The Mechanisms of Muscle Hypertrophy and Their Application to Resistance Training. J. Strength Cond. Res..

[43] Antonios T., Adds P.J. (2008). The Medial and Lateral Bellies of Gastrocnemius: A Cadaveric and Ultrasound Investigation. Clin. Anat..

[44] Edgerton V.R., Smith J.L., Simpson D.R. (1975). Muscle Fibre Type Populations of Human Leg Muscles. Histochem. J..

[45] Aagaard P. (2003). Training-Induced Changes in Neural Function. Exerc. Sport Sci. Rev..

[46] Trappe T.A., Raue U., Tesch P.A. (2004). Human Soleus Muscle Protein Synthesis Following Resistance Exercise. Acta Physiol. Scand..

[47] Kassiano W., Costa B., Kunevaliki G., Soares D., Stavinski N., Francsuel J., Carneiro M.A.S., Tricoli I., Nunes J.P., Ribeiro A.S. (2023). Muscle Swelling of the Triceps Surae in Response to Straight-Leg and Bent-Leg Calf Raise Exercises in Young Women. J. Strength Cond. Res..

[48] Finni T., Hodgson J.A., Lai A.M., Edgerton V.R., Sinha S. (2003). Nonuniform Strain of Human Soleus Aponeurosis-Tendon Complex during Submaximal Voluntary Contractions in Vivo. J. Appl. Physiol. (1985).

[49] Hodgson J.A., Finni T., Lai A.M., Edgerton V.R., Sinha S. (2006). Influence of Structure on the Tissue Dynamics of the Human Soleus Muscle Observed in MRI Studies during Isometric Contractions. J. Morphol..

[50] Brughelli M., Cronin J., Levin G., Chaouachi A. (2008). Understanding Change of Direction Ability in Sport: A Review of Resistance Training Studies. Sports Med..

[51] Douglas J., Pearson S., Ross A., McGuigan M. (2017). Chronic Adaptations to Eccentric Training: A Systematic Review. Sports Med..

[52] Almutairi M., Hunter G., Lein D., Kim S., Bryan D., Inacio M., Hurt C., Reed W., Singh H. (2023). Enhancement of Muscle Shortening Torque Preloaded with Muscle Lengthening Is Joint-Specific. J. Hum. Kinet..

[53] Suchomel T.J., Nimphius S., Bellon C.R., Stone M.H. (2018). The Importance of Muscular Strength: Training Considerations. Sports Med..

[54] DeLeo J.M., Wolf A., Philipp N.M., Ackerman K.E., Fry A.C. (2025). The Relationship between Countermovement Jump Force-Time Characteristics and 2000-m Rowing Ergometer Performance. Front. Sports Act. Living.

[55] Behm D.G., Konrad A., Nakamura M., Alizadeh S., Culleton R., Hadjizadeh Anvar S., Pearson L.T., Ramirez-Campillo R., Sale D.G. (2025). A Narrative Review of Velocity-Based Training Best Practice: The Importance of Contraction Intent versus Movement Speed. Appl. Physiol. Nutr. Metab..

[56] Cormie P., McGuigan M.R., Newton R.U. (2011). Developing Maximal Neuromuscular Power: Part 2—Training Considerations for Improving Maximal Power Production. Sports Med..

[57] Zhu Z., Wu H., Li L., Jia M., Li D. (2024). Effects of Diverse Resistance Training Modalities on Performance Measures in Athletes: A Network Meta-Analysis. Front. Physiol..

[58] Cao S., Wang Z., Guo J., Geok S.K., Sun H., Liu J. (2024). The Effects of Plyometric Training on Physical Fitness and Skill-Related Performance in Female Basketball Players: A Systematic Review and Meta-Analysis. Front. Physiol..

[59] Berriel G.P., Schons P., Costa R.R., Oses V.H.S., Fischer G., Pantoja P.D., Kruel L.F.M., Peyré-Tartaruga L.A. (2021). Correlations Between Jump Performance in Block and Attack and the Performance in Official Games, Squat Jumps, and Countermovement Jumps of Professional Volleyball Players. J. Strength Cond. Res..

[60] Pawlik D., Mroczek D. (2023). Influence of Jump Height on the Game Efficiency in Elite Volleyball Players. Sci. Rep..

[61] Behringer M., Vom Heede A., Yue Z., Mester J. (2010). Effects of Resistance Training in Children and Adolescents: A Meta-Analysis. Pediatrics.

[62] Lloyd R.S., Oliver J.L. (2012). The Youth Physical Development Model: A New Approach to Long-Term Athletic Development. Strength Cond. J..

[63] Moran J., Sandercock G., Ramirez-Campillo R., Clark C.C.T., Fernandes J.F.T., Drury B. (2018). A Meta-Analysis of Resistance Training in Female Youth: Its Effect on Muscular Strength, and Shortcomings in the Literature. Sports Med..

[64] Du H., Liu S., Li M., Zhao K., Jiang W., You T., Wang Z., Zou D., Shu J., Liu C. (2025). Effects of Nutritional Supplements on Explosive Lower Limb Performance in Volleyball Players: A Systematic Review and Network Meta-Analysis. Nutrients.

[65] Wang Z., Du H., Li H., Zhao K., Zhao B., Ma Y., Zhang J., Wu K., Jiang W., Liu C. (2025). Effects of the Combined Supplementation of Caffeine and Rhodiola Rosea with Resistance Training on Lower Limb Explosive Power in Male Volleyball Players. Nutrients.

